# Open Versus Endoscopic Osteotomy of Posterosuperior Calcaneal Tuberosity for Haglund Syndrome: A Retrospective Cohort Study

**DOI:** 10.1177/23259671211001055

**Published:** 2021-04-19

**Authors:** Yanbin Pi, Yuelin Hu, Qinwei Guo, Dong Jiang, Xin Xie, Feng Zhao, Linxin Chen, Yingfang Ao, Chen Jiao

**Affiliations:** * Institute of Sports Medicine, Beijing Key Laboratory of Sports Injury, 66482 Peking University Third Hospital, Beijing, People’s Republic of China.; *Investigation performed at the Institute of Sports Medicine, Beijing Key Laboratory of Sports Injury, Peking University Third Hospital, Beijing, People’s Republic of China*

**Keywords:** Haglund deformity, endoscopic procedure, calcaneoplasty, retrocalcaneal impingement

## Abstract

**Background:**

Although endoscopic calcaneoplasty and retrocalcaneal debridement have been extensively applied to treat Haglund syndrome, evidence of the value of the endoscopic procedure remains to be fully established.

**Purpose/Hypothesis:**

The purpose of this study was to compare the postoperative outcomes and the amount of osteotomy between open and endoscopic surgery for the treatment of Haglund syndrome. It was hypothesized that endoscopic calcaneoplasty would lead to higher patient satisfaction and lower complication rates compared with open surgical techniques.

**Study Design:**

Cohort study; Level of evidence, 3.

**Methods:**

The following postoperative outcomes were compared between the open surgery group (n = 20) and the endoscopic surgery group (n = 27): visual analog scale for pain, American Orthopaedic Foot & Ankle Society ankle-hindfoot scale, Foot Function Index, Tegner score, Ankle Activity Score, and 36-Item Short Form Health Survey; postoperative complications; and duration of surgery. To determine the extent of resection, the authors compared the calcaneal height ratio, calcaneal resection ratio, calcaneal resection angle, pitch line, and Haglund deformity height between groups. The learning curve for endoscopic calcaneoplasty was also calculated.

**Results:**

There were no significant differences between the open and endoscopic groups on any outcome score. Two patients in the open group reported temporary paresthesia around the incisional site, indicating sural nerve injuries; no complication was reported in the endoscopy group. None of the parameters for extent of resection were statistically significant between the groups. The duration of surgery was 44.90 ± 10.52 and 65.39 ± 11.12 minutes in the open and endoscopy groups, respectively (*P* = .001). Regarding the learning curve for endoscopic calcaneoplasty (6 surgeons; 27 follow-up patients; 9 patients lost to follow-up), the duration of surgery reached a steady point of 55.68 ± 4.19 minutes after the fourth operation.

**Conclusion:**

The results of this study indicated that the endoscopy procedure was as effective as the open procedure. The endoscopic procedure required significantly more time than the open procedure, and the duration of the endoscopic procedure was shortened only after the fourth operation, suggesting that it requires high technical skills and familiarity with the anatomic relationships.

Haglund syndrome is a chronic disorder with the following signs and symptoms: posterior heel pain, swelling, and morning stiffness. It is pathologically characterized by posterosuperior calcaneal prominence (Haglund deformity), retrocalcaneal bursitis, and insertional Achilles tendinopathy. It usually affects middle-aged women and has bilateral involvement. Numerous factors are associated with Haglund syndrome, including Achilles tendon contracture, rearfoot equinus, compensated rearfoot varus and forefoot valgus, rigid plantarflexed first ray, cavus foot, and trauma to the apophysis in childhood.^12^ Nevertheless, abnormal enlargement of the posterosuperior calcaneal prominence (Haglund deformity) impinging on the distal Achilles tendon is considered the most critical anatomic factor in Haglund syndrome. As the abnormal impingement continues, Achilles tendinitis may occur with tendon degeneration or rupture.^8,18^

Although response to nonoperative treatment is lower in impingement tendinitis than in noninsertional Achilles tendinopathy, nonoperative treatment should always be performed before considering surgery. The goal of nonoperative treatment is to reduce inflammation of the retrocalcaneus and relieve posterior heel pain. The nonoperative treatment strategy includes reduction in activity intensities, nonsteroidal anti-inflammatory drug administration, ice bag compression, footwear modification, gastrocnemius stretch, extracorporeal shock wave therapy, and injections with corticosteroid or platelet-rich plasma.^13^

If nonoperative treatment fails, surgical intervention with endoscopic calcaneoplasty or retrocalcaneal debridement is a reasonable choice. Complications have been reported in open retrocalcaneal debridement,^26^ including wound problems, infection, scar irritation, paresthesia, Achilles tendon lesion, and deep vein thrombosis. Endoscopic calcaneoplasty through medial and lateral portals with the patient in the prone position was first reported by van Dijk et al^24^ in 2001. Thereafter, the endoscopic procedure, which showed higher patient satisfaction and lower complication rates than open surgical techniques, has become increasingly applied.^5,20,25^

Compared with open procedures, endoscopic calcaneoplasty has the following potential advantages: minimal incision with a low rate of wound problems, shorter recovery time, and precise decompression of impinged calcaneal tuberosity.^5,24^ Moreover, the procedure prevents overresection and injuring the Achilles tendon insertion. Although this technique has been attempted in Haglund syndrome with varying extents of Achilles tendon injury, relative contraindications exist, including extensively degenerative Achilles tendon (>50%), large ossification, and bony spur requiring open debridement and reattachment of the Achilles tendon insertion.^11^

Nevertheless, endoscopic surgery is a technically challenging and time-consuming procedure, as it requires access to and visualization and removal of adequate bone from the calcaneal tuberosity, especially from medial to lateral corners. Previous studies have reported that resection of a sufficient amount of bone is essential for a good outcome.^1,3,6,7,10,16,21,24^ However, no clear guideline on the amount of bone that needs to be excised during calcaneoplasty has been established, and underestimation of bony volume to be excised may result in persistent posterior heel pain. Thus, identification of the location of impingement and determination of the extent of excision are technically challenging.^13^

Our study aimed to compare the postoperative outcomes and the amount of osteotomy between open and endoscopic surgery to clarify the value of an endoscopic procedure for the treatment of Haglund syndrome. We hypothesized that endoscopic calcaneoplasty would result in higher patient satisfaction and lower complication rates compared with open surgical techniques.

## Methods

### Study Design

**Figure 1.**
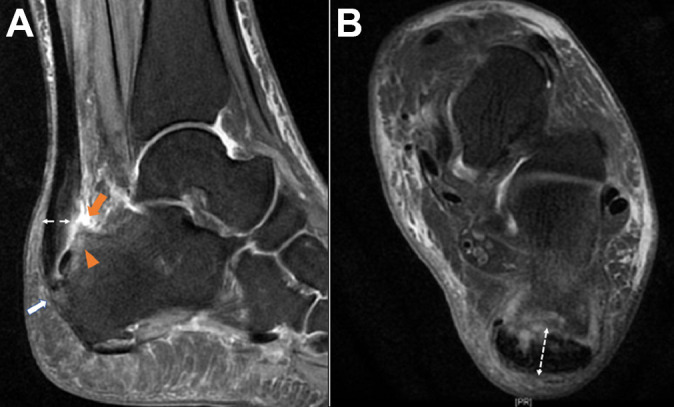
Signs of Haglund syndrome on (A) sagittal and (B) axial magnetic resonance imaging: retrocalcaneal bursitis (orange arrow), bone marrow edema (orange arrowhead), bony spur (white arrow), and Achilles tendon diameter (double-headed dashed arrow in both images). The intratendinous hyperintensity of the Achilles tendon in this patient was classified as Pomranz grade 1b.

All study protocols were approved by the local ethics committee. A total of 74 patients with Haglund syndrome who had calcaneoplasty from June 2015 to June 2019 were included in this retrospective study. The inclusion criteria were posterior heel pain and swelling, as well as the following characteristics shown on magnetic resonance imaging (MRI)^19,22,23^: retrocalcaneal exudation or bursitis, heterogeneous intratendinous hyperintensity <50% (grade 0, 1a, or 1b according to the Pomranz classification^15^), bone marrow edema in the posterosuperior calcaneal tuberosity, bony spurs on the Achilles insertion, and anteroposterior Achilles tendon thickness of 2 cm above the insertion measured in the horizontal view.^14,22^ In addition, the MRI had to be at least 6 months after the failed nonoperative treatment. The exclusion criteria were severe traumas or fractures, previous Achilles tendon surgery, Achilles tendon degeneration >50% (grade 2 or 3 by Pomranz classification) requiring reattachment or an augmentation procedure, congenital deformities, or ankle infection. Figure 1 shows the MRI characteristics of Haglund syndrome in a study patient.

**Figure 2.**
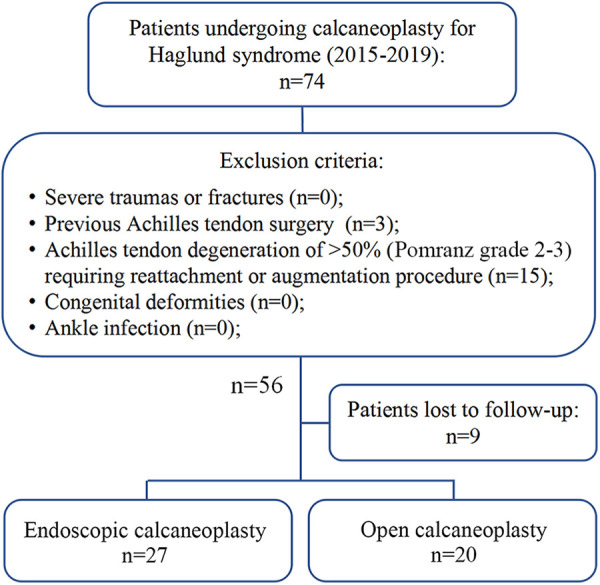
Study flowchart.

After applying the inclusion and exclusion criteria, 47 patients were included and were divided into 2 groups according to the preference of surgeons and patients: the endoscopic surgery group (n = 27) and the open surgery group (n = 20) (Figure 2). The patients’ characteristics, including age, sex, body mass index, time of follow-up, symptom onset, and history of corticosteroid administration, were recorded.

### Surgical Procedures and Postoperative Rehabilitation

All surgeries were performed according to previous studies^10,11,13^ by 6 subspecialists in ankle and foot surgery (Y.P., Y.H., Q.G., D.J., X.X., C.J.) at a single institution.

#### Open Calcaneoplasty

The patients were placed in the prone position, and a thigh tourniquet was inflated after exsanguination. A 4-cm longitudinal incision was made lateral to the Achilles tendon. The Achilles tendon was debrided and retracted posteriorly to expose the retrocalcaneal bursa. The inflamed bursa and the posterosuperior process of calcaneal tuberosity were excised by a bony chisel. If desired by the surgeon, fluoroscopy was performed to assess the resected amount of the calcaneal process. At the end of the procedure, the incision was closed in layers and compressive dressing was applied.^9^

#### Endoscopic Calcaneoplasty

**Figure 3.**
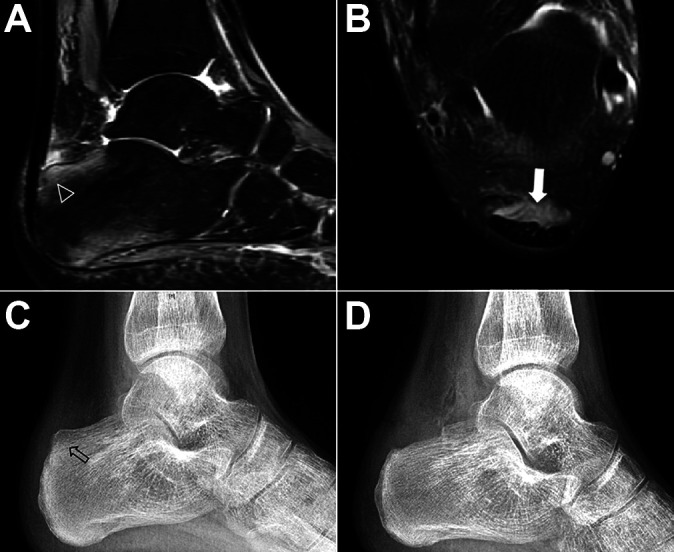
Endoscopic procedure for Haglund syndrome. (A and B) Retrocalcaneal impingement as visualized on magnetic resonance imaging. (A) Sagittal view with bone marrow edema (arrowhead). (B) Axial view with retrocalcaneal bursitis (white arrow). (C and D) Lateral ankle radiographs (C) with enlargement of posterosuperior calcaneal tuberosity with Haglund deformity (arrow) and (D) after posterosuperior calcaneal tuberosity was excised.

The patients were placed in the prone position. The portals were placed just lateral and medial to the Achilles tendon, as close as possible to the superior edge of the calcaneus, and as far posterior as possible. Retrocalcaneal bursitis with fibrosis was resected with a 4-mm resector/shaver. The bony landmarks of the prominence of calcaneal tuberosity were identified clearly. Calcaneal exostosis was exposed and resected with a 4-mm bur. Resection started at 1 edge (medial or lateral), was performed as far inferior as possible, and was completed at the insertion site of the Achilles tendon. Moreover, special attention was needed to remove the medial and lateral edges of the calcaneus and to smooth the edges carefully to prevent leaving bony prominences that might lead to clinical symptoms. Fluoroscopy could be performed to verify the amount of the calcaneal tuberosity removed. If the Achilles tendon was degenerated, debridement of the anterior part of the tendon was performed arthroscopically until it showed a normal color and texture. For the posterior part of the tendon involved, a percutaneous longitudinal split was made in the tendon by a scalpel to revasculate the degenerative tendon. Subsequently, the skin was carefully closed with 1 or 2 sutures at each incision. A compressive dressing was placed on both sides of the Achilles tendon.^6^ Imaging details are shown in Figure 3.

#### Rehabilitation

The rehabilitation protocol was the same for both groups. A plastic brace was applied to immobilize the ankles in the plantarflexion position. At 2 weeks postoperatively, a removable walking boot was used, weightbearing walking was started gradually, and passive and active range of motion were performed for 2 to 4 weeks postoperatively depending on the extent of Achilles tendon debridement. At 4 to 6 weeks after the operation, the patients were allowed to walk without restrictions. At 8 to 12 weeks postoperatively, the patients were able to return to their preoperative activities fully unrestricted.

### Clinical Outcomes

Clinical outcomes were evaluated using several clinical scoring systems^2,4,18^: the visual analog scale for pain, American Orthopaedic Foot & Ankle Society ankle-hindfoot scale, Foot Function Index, Tegner score, Ankle Activity Score, and 36-Item Short Form Health Survey.^4^ Complications were also documented and included the following: Achilles tendon avulsion, persistent posterior heel pain, wound problems, scar hyperesthesia, sural nerve injuries, and ankle stiffness.^13^ Operation duration was measured from skin incision to wound closing and compared between the groups.

The learning curves of the 6 surgeons for performing endoscopic calcaneoplasty were also recorded.

### Radiographic Evaluation

**Figure 4.**
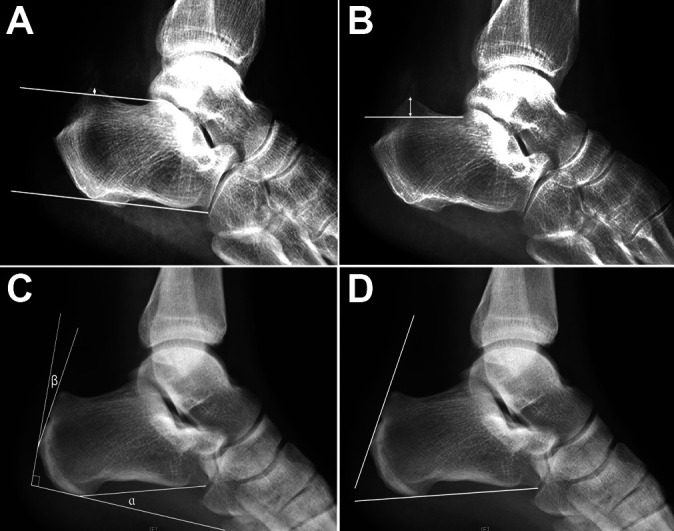
Characteristics of Haglund deformity as measured on preoperative lateral ankle radiographs. (A) The parallel pitch lines were obtained by drawing the inferior line from the inferior margin of the calcaneocuboid joint to the plantar tuberosity of the calcaneus; the superior line was drawn parallel to the inferior line beginning at the posterior margin of the subtalar joint. If the posterior calcaneal prominence was located above the superior line (arrow), it was considered abnormal and consistent with Haglund deformity. (B) The Haglund deformity height was obtained by drawing a line at the base of the posterosuperior calcaneal prominence and measuring the height of Haglund deformity perpendicular to that line (double-headed arrow). (C) The Chauveaux-Liet angle was measured as (α – β), where α is the inclination angle and β is the posterior angle of the calcaneus. (D) The Fowler-Philip angle was measured between an inferior line that was tangent to the inferior margin of the calcaneocuboid joint and the plantar tuberosity of the calcaneus and a superior line that was tangent to the posterior prominence at the insertion of the Achilles tendon.

Radiographic parameters were measured on lateral ankle radiographs taken preoperatively to determine the characteristics of Haglund deformity in each group, including the pitch line, Haglund height, Chauveaux-Liet angle, and Fowler-Philip angle^8,23^ (Figure 4).

**Figure 5.**
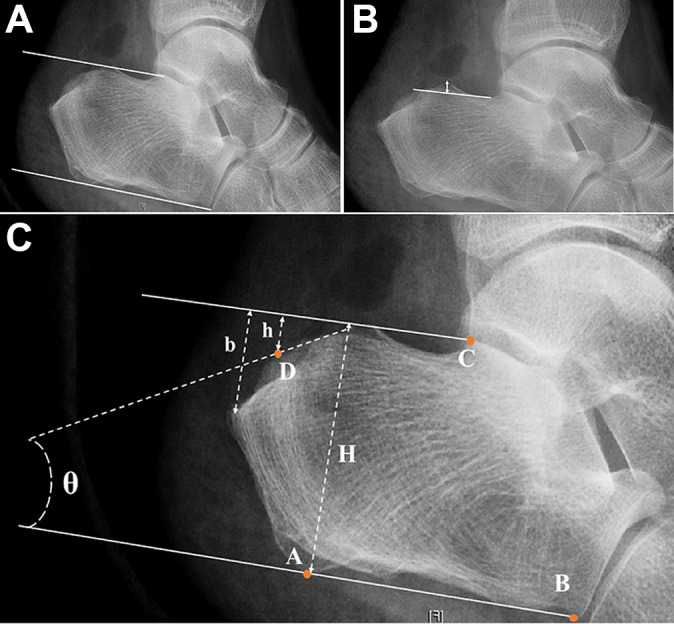
Radiographic parameters on postoperative radiographs. (A) Pitch line. (B) Haglund deformity height (double-headed arrow). (C) The calcaneal height ratio (CHR), calcaneal resection ratio (CRR), and calcaneal resection angle were measured as follows. The following landmarks were identified: the most inferior point of the posterior tuberosity, *A*; the inferior edge of the anterior calcaneus at the calcaneocuboid joint, *B*; the inferior parallel pitch line, line *AB*; the most posterior extension of the superior pitch line in panel (A), *C*; the exit point of the osteotomy from the posterior calcaneal tuberosity, *D*; the distance from the superior pitch line to the point of Achilles insertion, *b*; and the distance from the superior pitch line to point *D*, *h*. These landmarks were used to calculate the CHR (ratio of *h* and *H*), CRR (ratio of *h* and *b*), and calcaneal resection angle (θ; between the inferior pitch line and a line of best fit along the calcaneal resection).

As the pitch line and Haglund deformity height changed after osteotomy, those 2 parameters were measured and compared between groups to evaluate the extent of calcaneal osteotomy (Figure 5, A and B). We also measured the calcaneal resection angle, calcaneal height ratio as a measure of the resection magnitude, and calcaneal resection ratio in both the endoscopy and the open groups (Figure 5C).^17^

The data were documented and averaged by 3 independent and experienced sports medicine surgeons (Y.P., D.J., X.X.) who were blinded to the clinical details of the patients.

### Statistical Analysis

To determine any difference between the endoscopy and open groups, the patients’ baseline characteristics, outcome scores, and radiological measurements were analyzed and compared by *t* test and Mann-Whitney *U* test. A *P* value <.05 was considered statistically significant. SPSS 19 software (IBM Corp) was used for statistical analysis.

## Results

The patient and MRI characteristics of the endoscopy and open groups are shown in Table 1. There were no significant patient or preoperative MRI differences between groups (Table 1).

**Table 1:** Patient and MRI Characteristics Between Groups*^a^*

	Endoscopy (n = 27)	Open (n = 20)	*P* Value
Patient characteristics			
Age, y	35.6 ± 12.8	38.5 ± 11.0	.617
Sex, male/female, n	21/6	15/5	.661
BMI	24.55 ± 2.03	25.42 ± 3.34	.524
Time of follow-up, mo	35.81 ± 19.48	41.35 ± 15.70	.090
Onset time of symptoms, mo	30.25 ± 20.23	29.57 ± 25.83	.518
Corticosteroid administration, %	9.52	9.68	.795
MRI characteristics			
Retrocalcaneal bursitis, n	25	16	.480
Pomranz classification, 0/1a/1b, n	7/10/10	7/7/6	.433
Bone marrow edema, n	26	15	.090
Calcification, n	3	5	.253
Bony spur, n	5	6	.461
Achilles tendon thickness, mm	8.47 ± 1.41	8.95 ± 1.49	.375

*^a^*Data are reported as mean ± SD unless otherwise indicated. BMI, body mass index; MRI, magnetic resonance imaging.

### Clinical Outcomes

**Figure 6.**
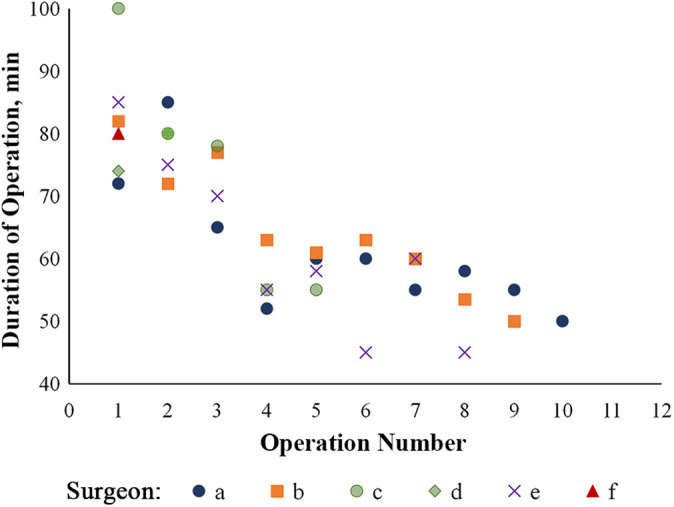
Learning curve for duration of operation according to number of operations performed for each of the 6 surgeons (represented by *a*-*f*) who performed endoscopic calcaneoplasty. a: solid blue dot; b: solid orange square; c: solid green dot; d: solid green diamond; e: purple cross; f: solid red triangle.

The comparison of clinical outcome scores in the endoscopy and open groups indicated no statistically significant differences between the groups (Table 2). Regarding complications, 2 patients in the open group reported temporary paresthesia around the incisional site, indicating sural nerve injuries; both fully recovered after 6 months of observation. No complications were reported in the endoscopy group. The operation duration was significantly different, at 65.4 ± 11.1 and 44.9 ± 10.5 minutes in the endoscopy and open groups, respectively (*P* = .001). The learning curve for endoscopic calcaneoplasty (27 follow-up patients; 9 patients lost to follow-up) of the 6 surgeons indicated that the duration of the operation reached a plateau of 55.7 ± 4.2 minutes after the fourth operation (Figure 6).

**Table 2:** Postoperative Clinical Outcomes in the Endoscopic and Open Groups*^a^*

	Endoscopy (n = 27)	Open (n = 20)	*P* Value
VAS-pain	1.5 ± 1.8	0.9 ± 1.2	.36
AOFAS	92.1 ± 8.0	96.1 ± 5.1	.22
FFI	3.7 ± 4.7	2.1 ± 2.7	.25
Tegner score	3.9 ± 1.9	3.2 ± 1.2	.32
AAS	5.0 ± 2.5	4.1 ± 1.6	.31
SF-36 domain			
PF	87.3 ± 13.2	86.5 ± 9.9	.87
RP	75.3 ± 26.9	72 ± 15.7	.72
BP	80.8 ± 18.0	78.9 ± 13.1	.77
GH	77.9 ± 19.7	84.3 ± 11.8	.34
VT	83 ± 13.7	84.6 ± 6.6	.76
SF	90.3 ± 10.3	96.6 ± 5.4	.10
RE	80.1 ± 23.9	73.8 ± 10.1	.40
MH	91.3 ± 14.0	96.8 ± 5.9	.33

*^a^*Data are reported as mean ± SD. AAS, Ankle Activity Score; AOFAS, American Orthopaedic Foot & Ankle Society; BP, body pain; FFI, Foot Function Index; GH, general health status; MH, mental health; PF, physical functioning; RE, role emotional; RP, role physical; SF, social functioning; SF-36, 36-Item Short Form Health Survey; VAS, visual analog scale; VT, vitality.

### Radiographic Evaluation

As assessed preoperatively, the Haglund deformity pitch lines were 68.42% and 65.39% (*P* = .804), the Haglund deformity heights were 8.79 ± 1.62 and 8.47 ± 1.61 mm (*P* = .577), the Chauveaux-Liet angles were 5.37° ± 8.38° and 5.93° ± 7.00° (*P* = .872), and the Fowler-Philip angles were 61.01° ± 5.41° and 63.03° ± 6.03° (*P* = .273) in the endoscopy and open groups, respectively. There were no statistically significant differences between the groups on any parameter.

The results of the postoperative measurements to determine the degree of osteotomy in the endoscopy and open groups are shown in Table 3. All parameters for the measurement of the amount of calcaneal osteotomy showed no statistically significant differences between the groups.

**Table 3:** Postoperative Radiological Measurement for Bony Resection in Endoscopic and Open Groups*^a^*

	Endoscopy (n = 27)	Open (n = 20)	*P* Value
Calcaneal resection angle (θ), deg	25.3 ± 11.7	27.9 ± 10.0	.60
Calcaneal height ratio	0.2 ± 0.1	0.1 ± 0.1	.26
Calcaneal resection ratio	0.5 ± 0.2	0.5 ± 0.2	.29
Postoperative pitch line, %	9.1	13.0	.55
Haglund deformity height, mm	5.0 ± 1.6	5.2 ± 2.7	.70

*^a^*Data are reported as mean ± SD unless otherwise indicated.

## Discussion

Haglund syndrome is a common cause of posterior heel pain. Pathological study has shown that enlarged calcaneal tuberosity impinges on the retrocalcaneal bursa and Achilles insertion during dorsiflexion of the foot, which results in retrocalcaneal bursitis and insertional tendinitis.^12^ Thus, the purpose of surgical treatment is resection of the prominence of calcaneal tuberosity and debridement of the retrocalcaneal bursa and Achilles tendon without weakening the Achilles tendon insertion. Such a treatment goal can be achieved by open surgical techniques and endoscopic surgery. Previous literature has reported that Haglund syndrome usually affects middle-aged women and has bilateral involvement, but according to our study, it is also common in young men. This result could be explained by more young men playing in demanding sports and willing to undergo surgical treatment.

Nevertheless, in the open procedure, an incision is made lateral to the Achilles tendons and adjacent to the sural nerve, which is vulnerable to injury. In our study, temporary paresthesia around the incision was reported in 2 patients who had an open procedure, which indicated sural nerve injuries, whereas no complication was reported in those who had an endoscopic procedure. This result suggested that endoscopic surgery is as effective as open surgery. Moreover, a comparison of postoperative outcomes and the amount of osteotomy between open and endoscopic surgery showed no significant differences.

However, an endoscopic procedure requires high technical knowledge, and the surgeons must be skillful and especially familiar with the anatomic relationships. In addition, evaluation of the learning curve of each surgeon revealed that a longer operative duration is required for endoscopic surgery compared with the open procedure (*P* = .001), and a C-arm fluoroscope may be needed intraoperatively to confirm the extent of endoscopic osteotomy. Moreover, the arthroscopic procedure costs more because the instrument used is not widely available. After the fourth operation, the operation duration was shortened to a steady point of 55.68 ± 4.19 minutes; nonetheless, such a duration is longer than that of the open procedure (44.90 ± 10.52 minutes).

Wiegerinck et al^26^ conducted a systematic review on endoscopic treatment of chronic retrocalcaneal bursitis. They evaluated 147 patients who underwent 150 procedures from 3 studies; the posterosuperior calcaneal process and retrocalcaneal bursa were resected in all patients. Postoperative outcomes were assessed, and they reported that 56% to 97% of patients rated their satisfaction level as excellent. The complication rate was extremely low, with only 1 major complication (0.7%) and 2 minor complications (1.3%). Moreover, most of the patients who underwent an endoscopic procedure had a smaller scar.

In our study, we included patients with Haglund syndrome and early-stage Achilles insertional tendinitis who only required retrocalcaneal debridement and calcaneoplasty. Those with Achilles tendon degeneration >50% (Pomranz grade 2 or 3) were excluded because they require more complicated surgical options, such as reattachment or an augmentation procedure, and we believe that an open procedure would be more reasonable and promising for such cases.

The largest limitations and weaknesses of this study are that it (1) had no randomization; (2) included the learning curve for the endoscopic procedure; (3) had no preoperative outcome measurements, so we were unable to assess improvement from the operation; (4) included no data on time to return to work or recreational activities; and generally (5) assessed a low-demand group, so we cannot be sure if 1 technique might be better in higher demand patients.

## Conclusion

Endoscopic calcaneoplasty is a technique that is as effective as the open procedure for Haglund syndrome and early-stage Achilles insertional tendinitis.
